# Nine quick tips for efficient bioinformatics curriculum development and training

**DOI:** 10.1371/journal.pcbi.1008007

**Published:** 2020-07-23

**Authors:** Susan McClatchy, Kristin M. Bass, Daniel M. Gatti, Adam Moylan, Gary Churchill

**Affiliations:** 1 The Jackson Laboratory, Bar Harbor, Maine, United States of America; 2 Rockman et al, San Francisco, California, United States of America; 3 College of the Atlantic, Bar Harbor, Maine, United States of America; Carnegie Mellon University, UNITED STATES

## Abstract

Biomedical research is becoming increasingly data driven. New technologies that generate large-scale, complex data are continually emerging and evolving. As a result, there is a concurrent need for training researchers to use and understand new computational tools. Here we describe an efficient and effective approach to developing curriculum materials that can be deployed in a research environment to meet this need.

## Introduction

Technological advances are driving exponential growth in biomedical data, prompting demand for training in new data analysis techniques. To keep pace, researchers must acquire the conceptual knowledge and practical skills needed to analyze large-scale, complex data. They must obtain this training while also carrying out their research, so the training must be of short duration, specific to their needs, and effective enough to apply in the near term. Absent relevant and immediately applicable training, biomedical researchers face significant barriers to progress and may be unable to adopt new technologies.

**Fig 1:**
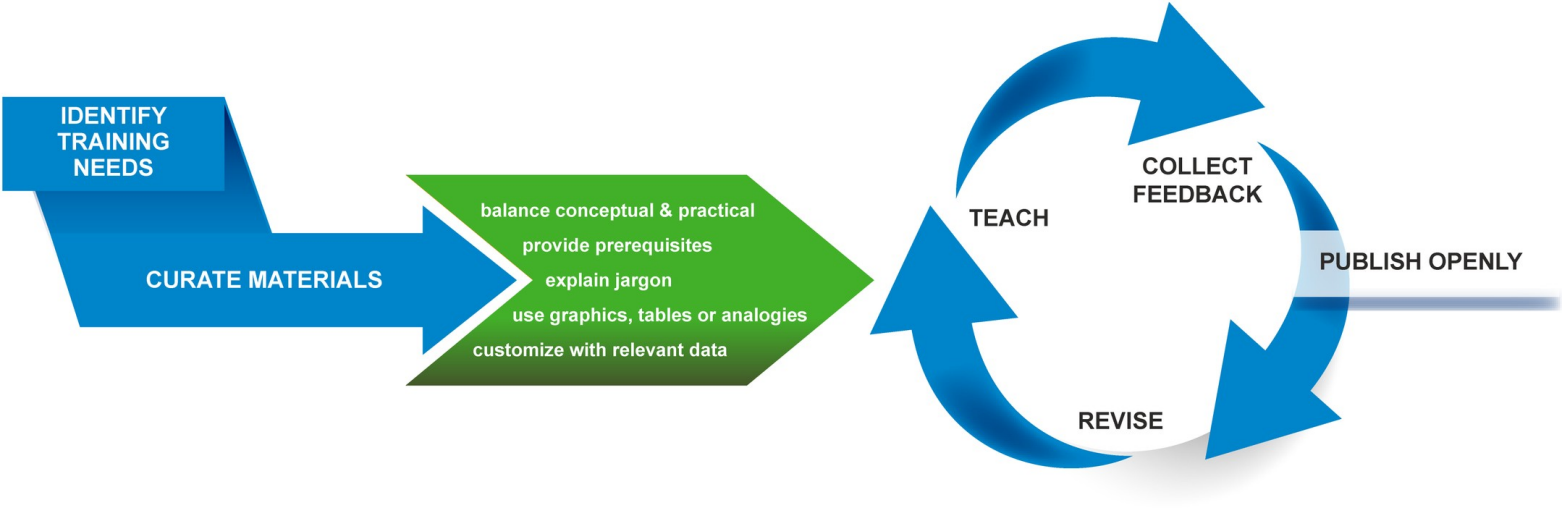
A process for efficient bioinformatics curriculum development and training. Effective training starts with identification of training needs. Existing materials that meet these training needs are curated and adapted to suit the audience. Adapted materials are tested and refined through teaching, feedback, and revision. The refined curricula are published openly for broad dissemination and reuse.

To provide relevant, up-to-date training, we have honed a strategy to develop curricula targeted to researchers’ specific needs and skill levels (Fig 1). Curriculum development is laborious, but it must be timely in order to meet training needs as they arise and to keep pace with technological change. The challenge is to rapidly develop and deploy training that combines conceptual knowledge with practical skills that can be applied by researchers. Our solution is to adapt open source materials, including software user guides and tutorials, that describe how to use software and to supplement these with conceptual information from open source texts and online lectures to provide understanding of the underlying analytical methods.

These 9 tips describe an efficient process for developing practical, hands-on courses for bioinformatics skill development. We start by identifying a critical training need. We then find and adapt open source software tutorials and other materials to develop customized training modules that can be produced and delivered without too great a lag time. Our preferred format is to develop a short series of hands-on workshops that can be completed in weekly sessions with durations of 2 to 4 hours. Although schedules can vary, we find that short sessions scheduled at a regular and convenient time maximize participation. We approach learning as a social activity [1] and prefer in-person group instruction with small groups of learners—although we have recently found that live online instruction can be effective. We have employed this process to create and deliver training sequences for biomedical researchers at the Jackson Laboratory and neighboring institutions. Our target audience for training is practicing biomedical researchers who are seeking to learn new data analysis skills. The tips in this article are designed for training program developers in research organizations, companies, and universities who provide training to build research workforce skills. There are many resources that address other aspects of bioinformatics curriculum development and provide complementary guidelines [2–5]. Our 9 tips outline a strategy for rapid deployment of effective skills development courses.

## 1. Identify critical training needs

To provide relevant and up-to-date training, it is important to identify critical training needs. The topic must be of interest to a broad enough audience to justify the effort needed to plan, prepare, and deliver. Training should address an immediate need and be directly applicable to ongoing research. We use several strategies to identify training needs and to define expected learning outcomes [6] and can perform a training needs analysis in less than 2 months. Importantly, training needs analysis centers attention on learners, rather than focusing on the instructor’s desire to teach a favorite topic. Training in basic skills is essential to provide a solid foundation for more advanced topics and should not be overlooked (see Tip 4).

To identify topics of broad relevance for biomedical researchers, we look for prevalent and persistent concerns such as those described in *Reproducibility Issues in Research with Animals and Animal Models* [7] and the *Nature* special collection *Challenges in Irreproducible Research* [8]. These publications describe performance gaps in research and suggest ways to resolve these gaps. We used these resources to design *Rigor and Reproducibility in Experimental Design* [9], a curriculum that addresses common mistakes in experimental design and provides training to support reproducible studies. Other resources, such as the *Nature* collection *Statistics for Biologists* [10] or articles like *Kick the Bar Chart Habit* [11], aim to correct common misconceptions in statistics and help to identify widespread training needs. The need for training in basic statistical methods motivated our development of *Statistical Inference for Biology* [12].

We evaluate organizational research strengths and strategic goals. Training that promotes an organization’s competitiveness or differentiates it from others should be prioritized to meet organizational needs [6]. For example, an organizational focus on genetic complexity motivated the development of *Quantitative Trait Mapping* [13], a curriculum that we adapted from a software user guide for the R package qtl2 (Karl Broman, https://kbroman.org/qtl2/) [14].

Self-organized interest groups provide a clear indication that there is enough interest to sustain a training effort. Participation in interest group meetings can help to gauge existing skills and identify knowledge gaps. Subject-matter experts can guide the design of training by identifying best practices and methods to address these gaps. We worked with a computational image analysis group and a domain expert who adapted a scikit-image tutorial [15] for image processing in Python [16,17]. Interest group members now support one another to sustain and grow the group’s skills and expertise by teaching introductory image analysis to new members and contributing to the development of advanced courses.

## 2. Curate existing materials to meet training needs

It is rarely necessary to build training materials from scratch. Once learning outcomes have been specified, the next step is to seek out existing training materials. Discussions with colleagues at conferences, courses, and workshops are good places to find out about training materials in specific topics. Digital repositories or collections such as the National Science Digital Library [18] and Multimedia Education Resource for Learning and Online Teaching [19] catalog open educational resources, many of which are aimed at graduate and professional levels. Openly licensed online books are an excellent resource for training. The *Python Data Science Handbook* [20], *Hands-on Programming with R* [21], *Data Analysis for the Life Sciences* [22], *R for Data Science* [23], and *Introduction to Data Science* [24] all have free versions available from the authors or publishers. High-quality training materials that can lead to desired training outcomes are in good supply.

It may be necessary to integrate materials from several sources. When compiling training material, it is important to discriminate between big ideas that are central to the topic, skills and knowledge that are critical and enabling, and those that are useful and should be familiar or accessible [5]. Prioritizing content around big ideas and critical skills is important for training that fits within tight schedules. Avoid the temptation to provide comprehensive coverage. Rather than cover lots of information, curricula should focus on core concepts and skills. Access to supporting material can be provided through links and references.

Here are 3 approaches to preparing relevant materials in order of increasing effort.

(1) Use existing materials that feature an applied approach. *R for Data Science* [23], for example, alternates between brief explanation, demonstration, and hands-on practice to teach R programming. Bioconductor publishes and updates training materials regularly [25]. This material can often be taught directly with little or no adaptation if your learners possess the computational and statistical background that they assume (see Tip 4).

(2) Adapt existing materials from user guides and software tutorials to address the learning goals. Add material to briefly introduce new concepts at the moment they are used in practical exercises (see Tip 6). Shorten practical exercises so that they can be executed during class sessions to promote feedback and understanding. Avoid longer exercises that require work outside of class time. If necessary, develop new short skills exercises. *Data Analysis for the Life Sciences* [22] is one example of material that can be tailored for short-term, intensive training for full-time researchers [12].

(3) Develop new materials only if necessary to meet a critical need for which no existing materials can be adapted. We produced *Rigor and Reproducibility in Experimental Design* [9] to address a critical need without adapting existing resources. For new materials or adaptations of existing ones, *Teaching Tech Together* [26] offers a chapter on lesson design and a lesson design template to guide curriculum development. *Understanding by Design* [5] provides a thorough treatment of the process of curriculum design.

Be mindful of licensing when reusing existing content (see Tip 9). Ideally, the content will carry an open access license that permits adaptation, such as some licenses provided by Creative Commons [27]. If not, request permission from the author or publisher before reusing or adapting content.

## 3. Balance the conceptual with the practical

Lecture and demonstration are good ways to teach people about concepts but are not good ways to teach people how to do things such as data analysis or coding. Practical skills are best learned in a hands-on setting, which requires guided practice and coaching [28,29]. When lecture is combined with guided practice, learners can develop desired skills grounded in understanding of larger ideas and concepts [5,30]. To be effective in preparing researchers, curriculum and training must balance practical training with conceptual understanding of new methods.

Software user guides and tutorials often explain the procedure for implementing a method with little detail of how the method works, when to use it, or what the results signify. Researchers can acquire procedural knowledge without understanding the underlying method and concepts that are needed to guide appropriate use and interpretation [31]. For example, a researcher can create a gene expression network in R by following the procedure detailed in a tutorial yet be unable to describe what it signifies or recognize when something has gone awry. Practical skills in the absence of conceptual understanding can produce flawed results.

Practical exercises may cover less content, but they provide experiences that support acquisition of new skills such as programming or data analytic techniques [28]. Ideally, practice will require the same skills that learners will need in their work and will be designed to be challenging [6].

A balance between the conceptual and the practical equips learners to perform tasks with a good understanding of what they are doing, why they are doing it, and what the results mean. Achieving this balance is often a matter of trial and error. One way to measure whether this balance has been reached is by teaching and collecting learner feedback (see Tip 8).

To illustrate a balance of conceptual and practical, a key idea in image analysis is that images are arrays that can be analyzed in many of the same ways as other types of data contained in arrays. We demonstrate this idea by analyzing image arrays using standard array manipulation methods, such as array slicing to crop an image. Familiarity with other tools for analyzing image data is helpful, but once the concept of images as arrays is familiar, learners are better prepared to understand and use other methods and software tools.

## 4. Provide materials for prerequisite knowledge and skills

One of the biggest challenges in developing training materials is to reach an audience with varied backgrounds and skill levels. Laying the groundwork with foundational training can help to bring all learners to the level of proficiency needed to tackle new material. When developing materials, look for the assumptions they make about prior knowledge, e.g., statistics or computer programming, as well as key biological concepts. Prerequisite knowledge and skills can be evaluated through carefully crafted screening questions. For example, a question that asks learners to rank their programming experience on a scale of 1 to 5 will indicate whether the course is a good fit for their skill level. Knowing your audience will help to determine which prerequisite knowledge and skills they will need [32,33]. Interview representatives of the target audience to identify where they might have difficulty with the materials. It can be helpful to consider learner personae that personify the target audience [3,26]. Don’t underestimate the need for prerequisite skills training. Careful attention to the preparation that learners need before they participate in training increases their likelihood of their success and the overall effectiveness of the training.

There are many resources available to address prerequisite knowledge and skills. We regularly offer Software Carpentry’s introductory Python and R training [34,35] to bring learners up to speed in programming skills needed to successfully participate in more advanced topics such as *Basic Image Analysis with Python* and *Quantitative Trait Mapping* [13,17]. Other resources for providing foundational training include *Hands-On Programming with R* [21], Quick-R [36], the swirl package for R [37], *Python Data Science Handbook* [20], and lessons in data organization and management from The Carpentries [38].

## 5. Explain technical terminology as it is introduced

Technical terminology, or jargon, can present a barrier to training across specialized fields of study. Modify teaching materials to explain technical terms or avoid them altogether by replacing them with clear, understandable language. Be especially mindful of acronyms, which should always be defined no matter how common or familiar they may seem. In addition, be aware of context-dependent definitions of common terms. For example, a vector to a mathematician is a one-dimensional array of numbers. In biology, a vector is a disease-transmitting organism like a mosquito, or a vehicle for transferring genetic material, like a plasmid. Differing meanings of common terms can cause confusion, so careful attention to meaning in context is warranted. A glossary is a helpful supplement for defining technical terms.

Recruit a partner who works in a different field of study to identify jargon or context-dependent terms. A nonexpert partner can help to translate the material by explaining the content in their own words. An expert then checks the translation for accuracy. Favoring the simpler, more direct language helps to circumvent the problem of expert blind spot—in which a highly knowledgeable instructor delves into technical details before addressing basic concepts [39]. We worked closely with a computer scientist who served as a nonexpert partner to help translate statistical concepts in *Data Analysis for the Life Sciences* [22], making them accessible to learners with little or no statistics background by rephrasing concepts with language that a nonstatistician might use [12].

## 6. Use graphics, tables, and analogies to introduce new concepts

Introducing new concepts at the moment when they are to be used in practice promotes knowledge retention [1,28]. Simple but precise graphics can be easily understood and reduce the time needed to introduce key concepts [40]. Many learners will appreciate and quickly grasp concepts presented in graphical form. Tables are another useful device that can be used to summarize large amounts of information that can be referred to later. Analogies provide conceptual models for acquiring new knowledge; they serve as memory aids and as devices for communicating complex ideas. Supporting graphics or tables can make content more accessible to those with certain conditions such as dyslexia, though conveying information with graphics alone excludes those with low vision or blindness [41,42]. Therefore, it is important to provide captions to ensure that graphics and tables provide complete and self-contained content. Attention to designing graphics and tables for accessibility benefits all learners, not only those with access needs.

**Fig 2:**
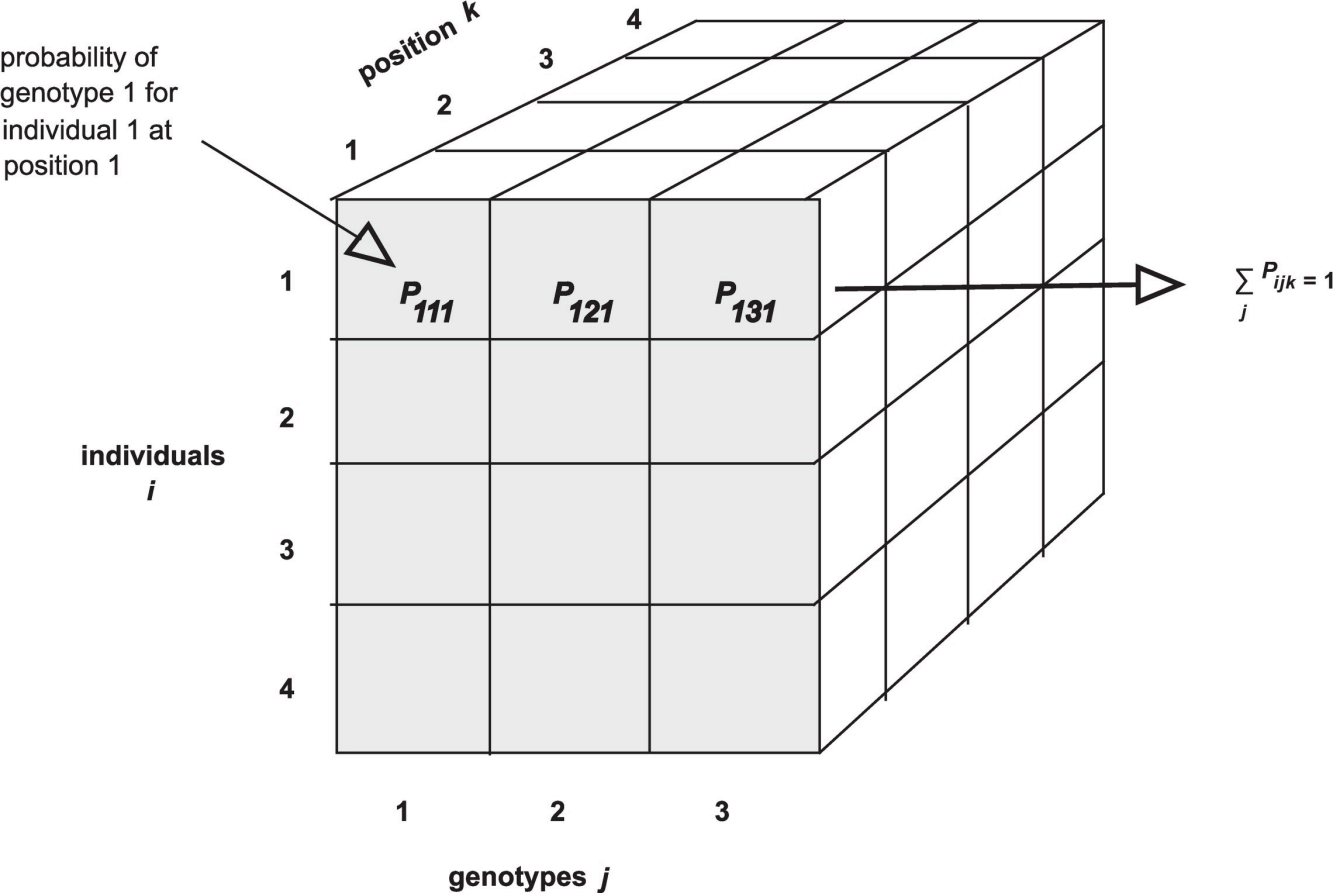
Simple graphics can make complex concepts more concrete and understandable. This example from Quantitative Trait Mapping illustrates a three-dimensional array of genotype probabilities across individuals, genotypes, and genomic positions. Each cell in the three-dimensional array represents a specific genotype in one individual at a genomic location. Uncertainty about genotypes is captured by probabilities that must sum to one at each location for each individual.

We introduce the concept of a genetic marker using the analogy of a landmark, like a building or a park with a numeric street address. We explain a genotype probability as a measure of the genotype between 2 adjacent landmarks (markers). Each individual has a genotype at every position in their genome. However, because we do not measure the genotypes between markers, there is uncertainty that we represent as a genotype probability. The graphic representation of genotype probabilities as a three-dimensional array (Fig 2) concisely represents a key data structure that stores genotype probabilities. We point out that the genotype probabilities at a given location for one individual should sum to one. We used this graphic to introduce the concept to learners in *Quantitative Trait Mapping* [13] immediately before they perform calculations on the genotype probabilities data structure.

## 7. Customize materials with data relevant to your audience

Learners will be more engaged when examples and practice data are related to their interests. Customize materials to the background of your learners by updating examples and exercises with relevant data that mirror their research. In our course on machine learning, we replaced housing price data in the scikit-learn tutorial [43] with protein expression data that are more familiar and relevant to biomedical researchers. It can sometimes be challenging to find and adapt data that work to illustrate key concepts. As an alternative to reworking examples and exercises, relevant data can be incorporated in a capstone exercise. Relevant data motivate researchers to learn and help them to transfer what they have learned directly to their own work.

## 8. Teach, collect feedback, revise, repeat

Frequent monitoring of learner feedback should be built into the curriculum. Incorporation of quick checks of understanding delivers information to both instructors and learners about what has or has not been understood and can indicate where adjustments are needed [44]. Assessments, such as brief written responses to a question or hand signals to indicate agreement or disagreement with a statement, reveal whether learners understand an idea or concept. Short practical exercises indicate whether learners are able to perform a specific task and provide valuable information about progress to both learners and instructors. A desired learning outcome in experimental design, for example, is the ability to calculate an appropriate sample size for an experiment. To address this training outcome, we ask learners to calculate power given sample sizes and statistical significance thresholds and describe the trade-offs between sample size, significance threshold, and cost. This brief exercise provides immediate feedback and an opportunity to correct misunderstandings. When practical exercises resemble tasks that learners would use in their work, retention and transfer of skills to work are enhanced [45].

Real-time assessment enables on-the-fly decision-making [46,47]. An instructor can choose to slow down and spend more time on a difficult topic or may move on to cover more material. We have adopted a practice featured in workshops offered by The Carpentries [38], providing students with 2 different-colored sticky notes that they can use to signal when they are falling behind or doing just fine. The same sticky notes are used to collect written feedback about the training at the end of each session. To evaluate the effectiveness of training in meeting learning goals, learners assess their own progress in pre- and postsession self-reports using structured questions that address their understanding of learning goals. Learners’ reactions, such as confidence in applying newfound skills or perceived usefulness of course content, can identify weaknesses and inform revisions needed to improve training.

Curriculum development should be an ongoing and iterative process that improves with each teaching event. The first teaching event might be bumpy and a bit disjointed, so choose a pilot audience that is hungry for learning, forgiving, and likely to provide useful critical feedback. Assessments drive the feedback loop between identification of critical training needs, curriculum design, and provision of training, which results in continuous improvement to training and positive response to training needs [5]. A rapid and robust feedback loop between instructors and learners allows information to flow in both directions, providing instructors with the opportunity to incorporate feedback and respond to learners’ needs during and after instruction.

In our first iteration of *Quantitative Trait Mapping* [13], learners were shown how to calculate the probabilities of genotypes at genomic locations between marker loci where the genotypes are measured. Feedback obtained at the end of the session indicated that learners did not understand why they were calculating genotype probabilities. In the next iteration, we prefaced the practical exercise with a short lecture on genetic markers and recombination. Following the exercise, we examined the results and discussed how they fit into the larger goal of mapping genetic loci that control complex traits. This provided the background knowledge and context they needed to apply the method in an informed way.

## 9. Make materials openly accessible for learners and other instructors

Following training, learners will need to review the material to reinforce their learning, to revisit concepts and skills not fully understood, and to access supplemental material. It is also helpful for learners to access training material beforehand so that they can preview content. Openly accessible materials can be adapted and taught by others, which has a multiplier effect for the reach of the work [48]. Provide materials in an easily accessible format such as a website or public repository. We use a Github template provided by The Carpentries to develop and publish lessons [49]. The template carries a Creative Commons Attribution license, which permits sharing and adaptation and requires appropriate attribution. The template design incorporates prerequisites, overarching questions, specific learning objectives, and exercises. As an example, we used this template to adapt parts of the book *Data Analysis for the Life Sciences* by copying openly licensed source files (R Markdown) for the book into the template. After modifying the source files, the template publishes a web page for each source file. The final result is published as a website [12]. Specific instructions for building a lesson and generating a website are provided in The Carpentries lesson template.

## Conclusion

We have described our process for producing up-to-date training materials in bioinformatics that is responsive to researchers’ needs and that is efficient to produce and deliver. We do so by identifying critical training needs and tailoring existing materials to meet these needs and produce desired learning outcomes. To tailor existing materials, we look for skills and prior knowledge they assume and supply background training to bring learners to the level needed to approach this new material. Training is organized around a few key concepts, with much class time devoted to applied practice that reinforces these concepts. We introduce concepts using simple graphics and analogies and define all technical terms and acronyms or replace them with more straightforward language. To motivate our learners and make training more relevant to their research, we customize training to use data sets from their field of study. We improve the curriculum through an iterative process of teaching, collecting feedback, and revising materials to iteratively improve results. Finally, we disseminate our materials as openly licensed websites and Github repositories so that others can access, teach, share, and adapt them as they see fit. This process efficiently creates training to provide researchers with the data analysis skills and knowledge they need to engage with new technologies. Effective training with timely delivery can open new avenues for inquiry and can drive successful and productive research.
